# The Requirements of Managing Phase I Clinical Trials Risks: The British and Italian Case Studies

**DOI:** 10.3390/epidemiologia5010009

**Published:** 2024-03-13

**Authors:** Davide Di Tonno, Laura Martena, Manuela Taurisano, Caterina Perlin, Anna Chiara Loiacono, Stefano Lagravinese, Santo Marsigliante, Michele Maffia, Susanna Esposito, Gianluca Villa, Giovanni Gori, Leonardo Bray, Alessandro Distante, Alessandro Miani, Prisco Piscitelli, Alberto Argentiero

**Affiliations:** 1 ClinOpsHub srl., 72023 Mesagne, Italy; davideditonno95@gmail.com (D.D.T.); lmartena@clinopshub.com (L.M.); mtaurisano@clinopshub.com (M.T.); cperlin@clinopshub.com (C.P.); aloiacono@clinopshub.com (A.C.L.); slagravinese@clinopshub.com (S.L.); 2 Department of Biological and Environmental Science and Technologies (Di.S.Te.B.A.), University of Salento, 73100 Lecce, Italy; santo.marsigliante@unisalento.it; 3 Department of Experimental Medicine, University of Salento, 73100 Lecce, Italy; michele.maffia@unisalento.it; 4 Division of Pediatrics, Department of Medicine and Surgery, University of Parma, 43121 Parma, Italy; susannamariaroberta.esposito@unipr.it; 5 Section of Anesthesiology, Intensive Care and Pain Medicine, Department of Health Sciences, University of Florence, 50100 Florence, Italy; gianluca.villa@unifi.it; 6 Clinical Trial Unit for Phase 1 Studies, Careggi University Hospital, 50100 Florence, Italy; 7 Clinical Pharmacology Center for Drug Experimentation, University Hospital of Pisa, 56126 Pisa, Italy; 8 School of Medicine, St. Camillus International University for Health Sciences, 00042 Rome, Italy; 9 Euro Mediterranean Scientific Biomedical Institute (ISBEM), 72023 Mesagne, Italy; distante@isbem.it (A.D.);; 10 Italian Society of Environmental Medicine, 20123 Milan, Italy; a.miani@simaitalia.org

**Keywords:** Phase 1 Clinical Trials, risk management, European Regulation, new drug development, UK, Italy

## Abstract

Phase I clinical trials represent a critical point in drug development because the investigational medicinal product is being tested in humans for the first time. For this reason, it is essential to evaluate and identify the Maximum Tolerated Dose (MTD) and the safety of the new compound. To mitigate the possible risks associated with drug administration and treatment, the European Competent Authority issued various guidelines to provide provisions and harmonize risk management processes. In the UK and Italy, particular attention should be paid to the Medicines & Healthcare Products Regulatory Agency (MHRA) phase I accreditation scheme and the specific rules set by the Italian Drug Authority through the AIFA Determination no. 809/2015. Both reference documents are based on the concept of quality risk management while conducting phase I clinical studies. Moreover, the AIFA determination outlines specific requirements for those sites that want to conduct non-profit phase I clinical trials. Indeed, the document reports peculiar activities to the “Clinical Trial Quality Team”, which is a team that should support the clinical site researchers in designing, starting, performing, and closing non-profit phase I studies. In this paper, we provide a general overview of the main European guidelines concerning the management of risks during phase I trials, focusing on the main peculiarities of the schemes and rules set by the MHRA and AIFA.

## 1. Phase I Clinical Trials: Shaping the Future of Medicine

Clinical trials are essential for drug development because new therapeutic strategies must be tested on humans to verify their safety and efficacy in real patient settings. Distinct phases of a clinical trial exist (from I to IV), each characterized by a variable duration and designed to meet specific endpoints. Pharmaceutical companies utilize data collected during clinical trials to request market access for new therapies.

A phase I clinical trial represents a crucial step in drug development, acting as a transition phase from pre-clinical to clinical studies [1]. In phase I studies, the drug candidates are administered for the first time on humans; for this reason, phase I studies are usually conducted on small groups of subjects, such as healthy volunteers [2,3] or patients affected by a well-defined disease. The primary aim of phase I studies is to evaluate the safety, tolerability, and pharmacokinetics of the new drug candidate and/or therapeutic strategy as well as the Maximum Tolerated Dose (MTD).

The first group of people included in the study (commonly identified as “cohort”) receives a low dose of the new drug/treatment and is observed very closely for a defined period. If the participants in the first cohort do not experience relevant side effects, the next cohort receives a higher dose. The process continues until the MTD is defined.

As few participants are involved in phase I clinical trials, rare relevant side effects may not be observed until more people receive the treatment (commonly in phases III or IV). Despite the potential risks related to the first-in-human administration of a newly developed drug, phase I clinical trials may represent the only remaining therapeutic chance for patients ineligible for current treatments. Indeed, people may join phase I clinical studies when all the other treatment options have proven ineffective [4,5,6]. In those cases, the expected benefits may exceed the potential risks of participating in the phase I study.

Due to the intrinsic characteristics of phase I clinical trials [7,8,9], competent authorities pay particular attention to patients’ safety and well-being. Various regulations and guidelines were released to protect phase I study participants from potential health risks.

## 2. The European Regulatory Framework

During the past few years, regulatory authorities have made tremendous efforts to mitigate the risks deriving from phase I studies by releasing guidelines to establish high-quality standards. Such guidelines were derived from disasters that occurred during the last 10 years (e.g., TGN-1412 trial) [10].

In July 2017, the European Medicines Agency (EMA) released its guideline regarding identifying and mitigating risks for phase I clinical trials [11]. The guideline applies to all chemical and biological investigational medicinal products (IMPs) but not to Advanced Therapeutic Medicinal products (ATMPs), which follow a different European legislation [12,13].

The first version of the European guideline provided provisions for the following:

1. Quality aspects of the IMP (e.g., determination of potency, strengths, materials used, etc.);
2. Pharmacokinetics, pharmacodynamics, and toxicology of the therapeutic agent candidate;
3. Dosing selection during the phase I clinical trial;
4. Phase I clinical studies management (planning, design, conduct).

Non-clinical data must support the design and application of First-In-Human (FIH) studies. The Investigator’s Brochure (IB) must report clinical (if present) and non-clinical data on the IMP, which should be updated when new data become available. The guideline mentioned above was updated and became effective in February 2018. This revision further assists the stakeholders in transitioning from non-clinical to early clinical development and identifying factors influencing risks for IMPs, such as the following:

1. Precautions to apply between treating subjects within a cohort as well as between cohorts and study parts;
2. Stopping rules;
3. Sponsor and investigator responsibilities.

In December 2009, the EMA published a guideline defining the “non-clinical safety studies for carrying out human clinical trials for pharmaceuticals” [14]. It provides recommendations on the following:

1. Pharmacological studies on the IMP;
2. Pharmacokinetics, pharmacodynamics, and toxicology;
3. Dose selection;
4. Safety tests.

Such studies should evaluate the effects of the therapeutic agent on the cardiovascular, respiratory, and central nervous systems. The studies should generally be conducted before human exposure and in accordance with the guidelines ICH S7A and ICH S7B.

As it is known, new drugs may include vaccines, whose development has been harmonized by the European guideline named “Guideline on Clinical Evaluation of Vaccines”, released in October 2006 and revised in 2023 [15]. This guideline provides recommendations on the following:

1. Immunogenicity, safety, and efficacy studies of vaccines;
2. Selection of the dose to be administered;
3. Concomitant medications;
4. Duration of the follow-up period;
5. Endpoint analysis;
6. Consideration of the particular population (e.g., pregnant women, immunodeficient subjects, etc.).

**Figure 1:**
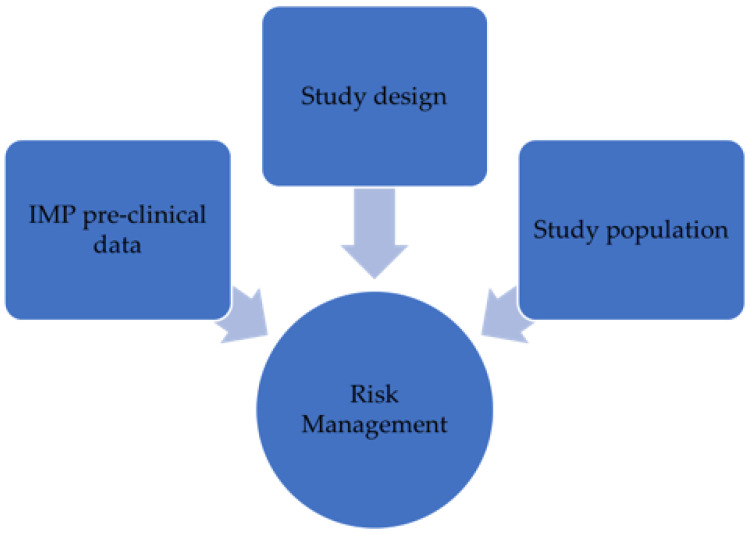
Factors influencing the risk management in phase I clinical trials.

As shown in Figure 1, the risk management system should evaluate the crucial elements when performing a clinical trial: IMP pre-clinical data, study design, and study population. These categories should be monitored during clinical development, and clinical trial features should be re-evaluated when new information related to the IMP becomes available through a risk assessment [16].

At the European level, it is interesting to note that Italy and the UK have published two similar reference documents to regulate the conduct of phase I clinical trials and safeguard the well-being of clinical trial participants, according to the Declaration of Helsinki and the Good Clinical Practice (GCP). The two countries issued the AIFA Determination no 809/2015 and the phase I accreditation scheme [17,18], respectively. Both documents set up the minimum requirements (structural and documental) that clinical centers should meet to conduct phase I clinical trials, and they have several common requirements, such as the following:

- The presence of a formal risk assessment/mitigation process of phase I clinical trials in place at the experimental center (SOPs, forms, etc.);
- The presence of trained personnel for medical emergencies;
- The structural requirements of phase I clinical units located in the hospitals.

To date, no other European countries issued similar regulations. For this reason, the article will further discuss the main characteristics of both reference documents.

## 3. MHRA Phase I Accreditation Scheme: The British Case Study

In April 2008, the Medicines & Healthcare Products Regulatory Agency (MHRA) published the phase I accreditation scheme, which is a guideline detailing quality and structural requirements for those clinical units performing FIH clinical trials in the UK [17]. The accreditation is voluntary, and originally, it was formally implemented for units conducting non-therapeutic phase I trials, including the units carrying out trials on healthy volunteers. The phase I accreditation scheme was not intended to cover phase I clinical trials in hospital settings (e.g., hospitalized oncological patients) as well as non-pharmacological or non-interventional drug trials.

The scheme initially classified the units into two types: standard accredited units (hospitals able to carry out all types of phase I clinical trials other than FIH trials) and supplementary accredited units (hospitals able to carry out clinical studies with compounds at all levels of risk). Over the years, the scheme has been revised to update the minimum compulsory requirements valid for the accreditation process, while it continues to be voluntary for units (both commercial and non-commercial) conducting phase I studies.

The phase I accreditation scheme provides a single system of classification based on the clinical trial unit’s facilities and procedures, including the training and experience of the unit’s personnel; therefore, the scheme allows for assessing the ability of the clinical trial unit to manage phase I trials, including those with certain risks. The voluntary accreditation does not cover the entire hospital system, all the wards and the personnel, or the phase I clinical trials conducted outside the phase I unit. Moreover, today the scheme covers early phase I clinical trials which involve healthy volunteers (HVs) and interventional phase I studies.

Serious Adverse Drug Reactions (SADRs) may occur in any clinical trial regardless of the perceived “risk” of certain compounds and molecules. There are also risks associated with the clinical study protocol itself (e.g., bronchoscopy, blood tests, etc.) and the possibility of observing reactions to commercialized drugs used as comparators. The phase I accreditation scheme crucially highlighted the importance of adequate personnel and facilities for dealing with any emergency.

As the accreditation scheme establishes requirements concerning the quality systems and management of a unit, selecting a phase I accredited unit guarantees compliance with basic regulatory requirements. The accreditation scheme also describes the responsibilities the sponsor should retain (e.g., pre-clinical data collection, analysis, and quality or phase I trial/dose escalation). Therefore, if the sponsor decides to perform the phase I clinical trial(s) using a phase I clinical trial unit compliant with the accreditation scheme, it must also meet any requirements specified by the accreditation scheme itself. Lastly, the sponsor should consider all the potential risks related to the clinical trials and mitigate them.

Hospitals that wish to apply for accreditation have to submit the following documents to the MHRA Good Clinical Practice Inspectorate:

1. A completed application declaration form;
2. A phase I accreditation compliance checklist;
3. Any associated documents.

Upon completing a successful verification of all the requirements, the inspectorate recommends the clinical trial unit for accreditation and issues a certificate valid for three years. The accredited unit must submit an updated phase I accreditation compliance checklist before each subsequent re-accreditation inspection or in the presence of significant changes.

These may include the following:

- Relocation of the unit or change in facilities;
- Significant changes to procedures that impact on key aspects of the accreditation scheme;
- Changes in key personnel (e.g., the medical director, any PIs authorized for FIH, senior nurses, the clinic manager, the pharmacist, and the Quality Assurance (QA) manager);
- Significant contractual changes in agreements with local hospitals;
- Significant changes in the clinical trial unit systems.

Once accredited, phase I clinical trial units must demonstrate continuous compliance with the requirements of the scheme to maintain accreditation. However, if serious issues are identified, they may lead to a temporary suspension; alternatively, the units may lose the accreditation status. When a unit is suspended or removed from the accreditation scheme, subjects’ recruitment and treatment are not compromised; nevertheless, it must inform the sponsors about their suspension or removal.

## 4. Regulatory Requirements of Managing Risks of Phase I Clinical Trials: The Italian Case Study

The Good Clinical Practice (GCP) guideline establishes that the sponsor is responsible for overseeing any trial-related activity (ICH-GCP R2 par. 5.2.2 *addendum*) [19]. Indeed, this aspect was also reported in Italian Legislative Decree 200/2007, at Art. 6, par. 3, stating that the sponsor has to guarantee that the clinical trial is conducted in compliance with GCP and the current legislation [20,21].

Unlike in the UK, the Italian experimental centers that wish to conduct phase I clinical trials must comply with the Italian Drug Authority (AIFA) Determination no 809/2015 and self-certify the possession of the requirements described in the above-mentioned determination to conduct phase I clinical trials. Self-certified centers are listed in a registry, which is regularly updated by the AIFA and published on the institutional website of the Italian Competent Authority.

The AIFA determination establishes specific standards such as the following:

- The presence of a medical director responsible for the activities carried out at the phase I unit;
- Structural requirements of the clinical site;
- Presence of a robust Quality Management System (QMS) to conduct phase I clinical studies.

As regards the structural requirements of the clinical site, this must possess appropriate areas where the subject can be monitored during the IMP administration. Equipment, crash carts, and gurneys must be maintained, and the maintenance process should be clearly described in SOPs. Lastly, the clinical personnel should be trained to handle any emergency.

The QMS should cover all the phase I processes at the clinical site, and a QA Manager should be appointed. The hospital personnel should be aware of the SOPs, and regular system audits must be performed to evaluate the compliance of the phase I unit with the national and international regulations. Study audits should be planned if phase I studies are ongoing.

The clinical sites must self-certify the possession of the above-mentioned requirements to the AIFA, which can inspect those sites to verify their compliance with the AIFA determination. Unlike in the UK, the self-certification of such requirements does not expire in Italy. The clinical sites can then proceed with the conduct of phase I clinical trials. However, as in the UK, any significant changes must be communicated to the AIFA by the medical director.

Interestingly, for managing non-profit phase I clinical studies, the Clinical Trial Quality Team project (promoted by the AIFA in 2006) played a crucial role [18].

The project was born to foster experimental sites to manage all non-profit trial-related aspects based on a Quality Management System and to guarantee the compliance of all activities to Good Clinical Practice and applicable legislation [12]. In this way, the Italian Competent Authority aimed to build up a framework of public hospitals that could stand out from others and demonstrate to sponsors their commitment to guaranteeing high-quality standards in conducting clinical trials. 

Characteristics, legal basis, and requirements to participate in the project are described in the AIFA document dated 2008 and subsequently updated in 2010.

The document highlights that the hospital top management should appoint a dedicated Clinical Trial Quality Team (CTQT) to help non-profit sponsor(s) and principal investigator(s) during the following:

- Trial start-up activities;
- Clinical trial monitoring;
- Clinical trial termination.

**Figure 2:**
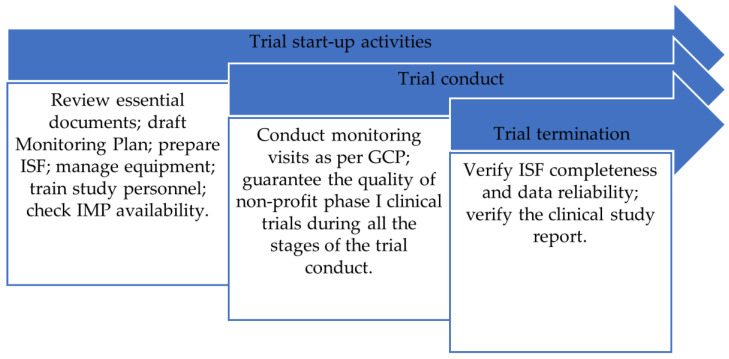
Main activities of the CTQT during non-profit phase I clinical trial management.

As shown in Figure 2, the CTQT should ensure, through defined processes, the overall quality of non-profit phase I clinical trial management.

## 5. Main Characteristics of the Clinical Trial Quality Team

As it is possible to understand, the CTQT functions substitute the industrial sponsor responsibilities as per the ICH-GCP (R2).

The AIFA document was soon after mentioned in AIFA Determination no. 809/2015 released by the Italian Competent Authority [17]. This determination and the AIFA document greatly impacted Italian experimental centers because the AIFA attributes to the CTQT the responsibility of managing all aspects of non-profit phase I clinical trials (such as start-up, conduct, and termination). This means that it is the responsibility of the non-profit sponsor to guarantee the compliance of the above-mentioned activities with the current legislation.

The possession of the requirements must be documented by sending a self-certification to the AIFA according to AIFA Determination no. 451/2016.

The CTQT should have the following:

- An organizational chart with enough qualified staff members;
- An internal regulation that explains the CTQT functions and roles;
- Standard Operating Procedures (SOPs) that cover every aspect of clinical trial management.

Based on the AIFA document, the CTQT can comprise internal personnel and consultants. People working on the project can be exclusively dedicated to it or perform various roles as well.

Remarkably, the CTQT personnel should demonstrate to have accomplished at least ten days of theoretical training before joining the team and should maintain educational qualifications by performing five days of refresher training every year.

At the beginning of the AIFA Determination no. 809/2015 implementation process, the experimental personnel had to integrate the requirements established by the Italian Competent Authority with Quality System guidelines of the hospitals’ quality offices (most of which based on UNI EN ISO 9001:2015). It required a great effort for public hospitals to comply with the national law and the ICH-GCP (R2) to act as sponsors for non-profit phase I clinical trials.

Moreover, most of the clinical sites’ personnel involved in the CTQT activities tried to set up a quality system composed of both internal regulations and SOPs. However, it is possible to find different organizational structures, such as the following:

- A CTQT quality system including internal regulations and the hospital SOPs which the quality team refers to;
- A CTQT quality system with dedicated internal regulations and SOPs;
- A CTQT quality system with dedicated internal regulations and dedicated/hospital SOPs to refer to. In this case, if no hospital SOPs describe the processes required by the CTQT document, new SOPs are set up.

Lastly, the CTQT composition may vary from one center to another. Indeed, it is possible to find a variety of organizational structures that can be summarized as follows:

1. Phase I unit-dedicated CTQT. In this type of organization, the quality team is exclusively dedicated to that specific phase I clinical unit, and it is responsible for the organization and for carrying out non-profit phase I clinical trials.
2. Hospital-derived CTQT. In this case, the quality team may supervise the organization of multiple phase I clinical trials within the same hospital but conducted at different clinical trial units.

## 6. Challenges and Opportunities of the Clinical Trial Quality Team

The main challenge encountered by the phase I clinical units is to be compliant with the compulsory training required by the AIFA documentation. As specified above, the CTQT personnel needs ten days of theoretical training before joining the CTQT and five days per year to maintain the qualification.

Another problem hospitals often face is providing a proper workload assessment to determine which and how much personnel should be involved in the project. Very often, the number of CTQT components does not correspond to the actual needs as there is not sufficient hospital personnel. However, sometimes, the non-profit phase I clinical trials can sometimes receive the support of CROs for clinical trial management. In this case, the latter acts as the CTQT, and the hospital must identify a person who can coordinate the different activities among the hospital, the non-profit sponsor, and the CRO (if present). The identified person should be familiar with the clinical studies management fundamentals and be able to supervise all activities.

Lastly, the company’s quality offices personnel often face difficulties integrating the requirements of the AIFA document with the company’s procedures, as these are often based on UNI EN ISO 9001:2015 standards, while the AIFA document is based on Good Clinical Practice. Therefore, it is necessary to have a significant understanding of the international standards to efficiently integrate them and to create a harmonized Quality Management System that can fit both clinical practice and clinical trial management, as it has been recognized in other research fields [22,23,24,25].

## 7. Final Remarks

To date, no official information about the number of hospitals with a CTQT is available.

Moreover, the latest decree published, namely the Italian Decree dated 31 December 2021 released by the Ministry of Health, remarked on the necessity of conducting clinical trials in experimental centers compliant with quality standards that must be set up by the Italian Competent Authority. As of today, it is unknown whether the requirements for phase I clinical units will also be extended to phase II and III clinical trials or re-evaluated and extended to studies of all stages.

## Data Availability

The data is available within the manuscript.
